# Predictors of self-reported hypertension among women of reproductive age in North Dakota

**DOI:** 10.1186/s12889-024-20525-6

**Published:** 2025-01-03

**Authors:** Corey A. Day, Grace Njau, Matthew Schmidt, Agricola Odoi

**Affiliations:** 1 Department of Biomedical and Diagnostic Sciences, College of Veterinary Medicine, University of Tennessee, Knoxville, TN USA; 2 Division of Special Projects & Health Analytics, North Dakota Department of Health, Bismarck, ND USA

**Keywords:** Reproductive age women, Hypertension, Predictors, Risk factors, Imputation, Logistic regression, Conceptual model, Behavioral risk factor surveillance system, North Dakota, United States of America, USA

## Abstract

**Background:**

Understanding the risk factors of hypertension among women of reproductive age (18–44 years) is important for guiding health programs aimed at reducing the burden of hypertensive disorders in this population. Therefore, the objective of this study was to investigate predictors of self-reported hypertension among women of reproductive age in North Dakota.

**Methods:**

Behavioral Risk Factor Surveillance System data for the years 2017, 2019, and 2021 were obtained from North Dakota Department of Health and Human Services. A conceptual model was used to identify potential predictors of hypertension including sociodemographic characteristics, behavioral factors, chronic health conditions, and healthcare access. A multivariable binary logistic regression model was then used to identify significant predictors of hypertension. The predictive ability of the final model was assessed using a Receiver Operating Characteristic (ROC) curve and area under the curve (AUC).

**Results:**

The odds of hypertension were significantly higher among women of reproductive age who reported frequent mental distress (odds ratio [OR] = 2.0, 95% confidence interval [CI] = 1.3–3.3), resided in a primary care health professional shortage area (OR = 1.8, 95% CI = 1.2–2.6), were obese (OR = 2.6, 95% CI = 1.7–4.1) and were 35–44 years old (OR = 2.3, 95% CI = 1.6–3.4), relative to their counterparts who did not have frequent mental distress, did not reside in a health professional shortage area, had a normal body mass index, and were 18–34 years old, respectively. Additionally, the odds of hypertension were lower among women who did not have a checkup within the last year compared to those who did have a checkup within the last year (OR = 0.6, 95% CI = 0.4–0.9). The AUC of the final model was 0.68.

**Conclusions:**

There is evidence that frequent mental distress and disparities in healthcare access or utilization are predictors of hypertension among women of reproductive age in North Dakota. Further research is warranted to determine whether improved mental health can reduce the risk of hypertension in this population. Public health officials may consider promoting hypertension awareness and control programs in areas with limited access to healthcare professionals.

## Background

Chronic hypertension among women of reproductive age is an important risk factor of hypertensive disorders of pregnancy, a leading cause of pregnancy-related mortality in the United States (US) [1–4]. Rates of perinatal mortality are significantly higher among pregnant women with chronic hypertension than those with normotensive pregnancies [5], and women who suffer hypertensive disorders of pregnancy have substantially higher risks of cardiovascular disease later in life [3, 6]. Rates of chronic and gestational hypertension are increasing in the US, due in part to increased prevalence of obesity and higher average maternal age [7, 8]. Consequently, there is growing awareness of the need for effective prevention and control of hypertension among women of reproductive age to improve women’s cardiovascular and maternal health outcomes [9, 10].

There are important demographic disparities in chronic hypertension risk among women across the US. Non-Hispanic Black and American Indian/Alaskan Native women are substantially more likely to develop chronic hypertension than White women [11, 12], and women residing in rural areas are more likely to develop chronic hypertension than those living in urban environments [13]. Sociodemographic factors represent additional disparities in hypertension prevalence, as women with lower income and education levels tend to have higher prevalence of hypertension than those with higher socioeconomic metrics [14]. Efforts to address these disparities are necessary to mitigate increasing rates of hypertension in the US [15].

North Dakota has large rural and substantial minority populations with severe disparities in health outcomes and healthcare access, which may contribute to disparities in hypertension awareness, prevention, and control [11–13]. Thirty-eight of the 53 North Dakota counties are designated by the Health Resources and Services Administration as primary care health professional shortage areas (HPSAs) [16]. Residents of HPSAs have lower access to medical care, including outpatient and preventive care, than people living in non-shortage areas [17, 18]. North Dakota also has substantial minority populations. Non-Hispanic American Indians are the most abundant minority ethnic/racial group, comprising more than 5% of the overall population [19]. The American Indian population in North Dakota faces severe disparities in general health status and adverse pregnancy outcomes relative to the general population, including higher infant mortality rates, lower access to satisfactory prenatal care, higher rates of gestational diabetes, and higher rates of adverse childhood experiences [20–24]. In the northern Great Plains region, which includes North Dakota, American Indian health outcomes tend to be substantially worse than among American Indian populations in the US overall, indicating that these racial disparities are heightened by conditions associated with this region [22, 23, 25].

There have been limited studies of disparities and predictors of chronic hypertension in North Dakota, especially among women of reproductive age. Therefore, the objectives of this study were to investigate predictors of self-reported chronic hypertension among women of reproductive age. The results of this study can be used to guide efforts to control pre-pregnancy hypertension and reduce the burden of hypertensive disorders among women of reproductive age in North Dakota.

## Methods

### Ethics approval

This study was reviewed by the University of Tennessee Institutional Review Board (Number: UTK IRB-24–08175-XM) and determined to be eligible for exempt review under 45 CFR 46.101. Anonymized secondary data were provided to researchers without identifiers. Study subjects were not re-identified or contacted by the investigators. All relevant guidelines and regulations were adhered to during the investigation.

### Study area and data source

This study was conducted in North Dakota, a state with 53 counties and a population of approximately 773,000 people. The state’s population is predominately non-Hispanic White (83%), followed by non-Hispanic American Indian (5%), Hispanic or Latino (4%), and non-Hispanic Black (3%). All other races or combinations of races comprise approximately 5% of the population. Women of reproductive age (18–44 years) comprise 18% of the total population [19].

Behavioral Risk Factor Surveillance System (BRFSS) data from the years 2017, 2019, and 2021 were obtained from the North Dakota Department of Health and Human Services (NDDHHS). The BRFSS is a telephone-based survey of health-related behaviors, chronic health conditions, and usage of medical services. The survey questionnaire is administered annually by the NDDHHS in partnership with the Centers for Disease Control and Prevention (CDC). Eligible participants include noninstitutionalized females of reproductive age (18–44 years). Questions relating to high blood pressure are administered on a bi-annual basis [26], so data on hypertension were not available in the 2018 or 2020 BRFSS data.

### Study population

The study population was women of reproductive age (18–44 years old) who responded to the question “Have you ever been told by a doctor, nurse, or other health professional that you have high blood pressure?”. Respondents who answered “Yes” were provided the follow-up question: “Was this only when you were pregnant?” Respondents who were never told they had high blood pressure, had borderline high blood pressure, or were only told they had high blood pressure during pregnancy were categorized as non-hypertensive. Those who said that they had been told they had high blood pressure were categorized as having hypertension.

### Selection of potential predictors

**Fig. 1:**
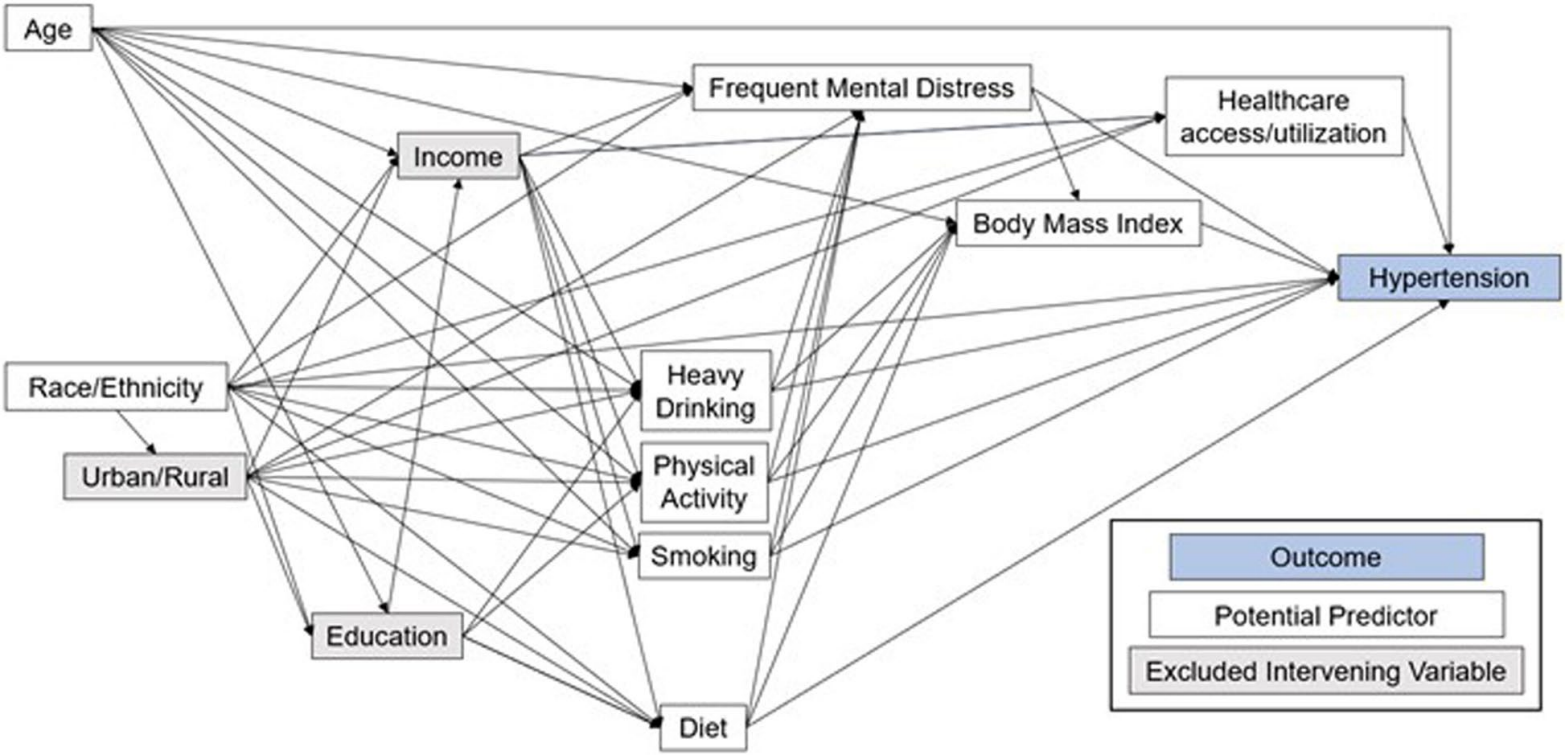
Conceptual model of predictors of hypertension among women aged 18–44 years in North Dakota

A conceptual causal model was developed based on biological knowledge and questions asked in the BRFSS survey (Fig. 1). The variables that were considered as potential predictors of hypertension are listed in Table 1 and consist of a variety of demographic factors, behavioral risk factors, chronic conditions, and variables associated with healthcare access and utilization including: age, race/ethnicity, smoking status, binge drinking, physical activity, fruit and vegetable consumption, frequent mental distress, body mass index (BMI), residence in an HPSA, inhibitive medical costs, personal healthcare provider, time of most recent checkup, and health insurance.

**Table 1:** Demographic factors, lifestyle characteristics, and chronic health status of women aged 18–44 years in North Dakota, 2017–2021

Category	Characteristic	Weighted Frequency (standard error)	Percent Hypertension (95% Confidence Level)
**Demographics**	**Age**		
	18–34	91,876 (3,138)	2.4 (2.4, 5.0)
	35–44	44,753 (1,750)	6.9 (6.9, 11.6)
	**Race**		
	White	107,025 (3,021)	5.1 (3.9, 6.2)
	American Indian	7,314 (788)	8.9 (3.1, 14.7)
	Black	6,042 (951)	6.7 (0, 13.6)
	Other Race/Ethnicity	15,912 (1,462)	11.9 (5.7, 18.1)
**Behaviors**	**Heavy Drinking**		
	> 7 drinks per week	10,268 (1,045)	4.4 (0, 9.8)
	≤ 7 drinks per week	119,186 (3,290)	5.8 (4.5, 7.0)
	**Smoking Status**		
	Current smoker	22,193 (1,518)	8.9 (5.6, 12.2)
	Non-smoker	110,211 (3,170)	4.9 (3.6, 6.2)
	**Physical Activity Outside of Current Job in Last Month**		
	No physical activity	33,036 (1,850)	7.1 (4.2, 10.0)
	Some physical activity	99,177 (3,016)	4.9 (3.6, 6.2)
	**Fruit and Vegetable Consumption**		
	< 5 per day	103,458 (3,083)	5.8 (4.4, 7.1)
	≥ 5 per day	22,363 (1,490)	5.6 (2.3, 8.9)
**Chronic Conditions**	**Frequent Mental Distress (FMD)**		
	≥ 14 poor mental health days	27,505 (1,791)	8.0 (4.5, 11.6)
	< 14 poor mental health days	107,976 (3,081)	4.6 (3.5, 5.8)
	**Unimputed Body Mass Index (BMI)**		
	Obese (BMI ≥ 30)	37,376 (1,875)	10.2 (7.2, 13.3)
	Overweight (25 < BMI ≤ 29)	31,441 (1,763)	4.5 (2.1, 6.9)
	Normal (18.5 < BMI ≤ 25)	49,988 (2,251)	3.0 (1.7, 4.4)
	Underweight (BMI ≤ 18.5)	2,555 (531)	0.6 (0, 1.6)
	Unknown/Missing	15,269 (1,204)	10.1 (5.2, 15.1)
	**Imputed BMI**		
	Obese (BMI ≥ 30)	41,229 (1,916)	14.3 (11.1, 17.6)
	Overweight (25 < BMI ≤ 29)	34,388 (1,802)	8.6 (5.5, 11.7)
	Normal (18.5 < BMI ≤ 25)	55,046 (2,301)	4.8 (3.2, 6.4)
	Underweight (BMI ≤ 18.5)	2,736 (534)	0.5 (0, 1.5)
**Healthcare Access/Utilization**	**Problems with Medical Cost in Last 12 Months**		
	Could not afford needed care	59,589 (2,325)	3.0 (1.6, 4.4)
	Afforded all needed care	76,672 (2,662)	7.4 (5.6, 9.2)
	**Personal Doctor**		
	No personal doctor	27,940 (1,826)	6.2 (3.0, 9.5)
	Has personal doctor	108,445 (3,058)	5.4 (4.1, 6.6)
	**Most Recent Checkup**		
	Longer than 1 year ago	43,216 (2,074)	4.3 (1.8, 6.7)
	Within last year (reference)	89,890 (2,887)	5.8 (4.5, 7.2)
	**Healthcare Insurance**		
	No healthcare insurance	11,192 (1,105)	6.9 (2.2, 11.7)
	Has healthcare insurance	112,371 (3,329)	9.1 (7.8, 10.6)
	**Primary Care Health Professionals Shortage Area (HPSA) Score**		
	Resides in HPSA	44,428 (1,884)	11.5 (8.9, 14.1)
	Does not reside in HPSA	92,202 (3,054)	7.7 (5.8, 9.5)

### Data Preparation

#### Re-coding of variables

All data cleaning and analysis were conducted using SAS Version 9.4 [27]. Age was categorized into a binary variable of ages 18–34 and 35–44 years, corresponding with evidence that pregnancy-related morbidity and mortality is significantly higher in women ages 35 years and older [28, 29]. Race and ethnicity were categorized as non-Hispanic white, American Indian, non-Hispanic Black, or Other Race/Ethnicity. Frequent mental distress (FMD) was calculated based on respondents’ answer to the question “how many days during the past 30 days was your mental health not good?”, using the CDC definition of 14 or more bad mental health days to define FMD [30]. Body mass index was calculated and categorized by CDC based on the height and weight of the respondent (BMI under 18.5 = underweight; between 18.5–24.9 = normal weight; 25.0–29.9 = overweight; 30.0 or greater = obese). Time of most recent checkup was categorized as having had a checkup within the last year or having a checkup more than one year ago. Residence in a primary care HPSA was dichotomized based on the primary care HPSA score of the county where the respondent resided, with any score > 0 being categorized as living in an HPSA. All other predictors were dichotomous, e.g., whether the respondent was a current smoker (yes/no), whether the respondent was a heavy drinker (yes/no).

#### Imputation

The percentage of missing data was investigated for each potential predictor variable. For any predictor with more than 5% missing data in the target population, the missingness was analyzed in relation to other predictor variables and the outcome variable using univariable logistic regression. BMI had 5.5% missing data and so was a candidate for imputation. If there was evidence of an association between a predictor’s missing data and the outcome or other predictor variables, the data were considered not missing completely at random. Responses that were not missing completely at random were imputed using the surveyimpute procedure in SAS using the hot-deck method with weighted selection [31, 32]. Imputation cells included covariates that were expected to be associated with the predictor being imputed based on the causal model (Fig. 1) and that had a simple association with hypertension (Table 2). Five donor cells were provided for each imputed response, and sampling weights for imputed values were divided by the number of donor cells. The imputation was implemented on the entire dataset, including males and people older than 18–44 years old.

**Table 2:** Results of univariable binary logistic regression models investigating simple associations between hypertension and potential predictors among women aged 18–44 years in North Dakota, 2017–2021

Predictor	Odds Ratio (95% Confidence Interval)	*p*-value
**Age**		** < 0.001**
35–44	2.3 (1.6, 3.3)	< 0.001
18–34	reference	
**Race**		** < 0.001**
White	2.5 (1.4, 4.5)	0.001
American Indian	1.7 (0.6, 4.6)	0.3
Black	1.6 (0.9, 3.0)	0.1
Other Race/Ethnicity	reference	
**Heavy Drinking**		0.2
> 7 drinks per week	0.7 (0.2, 1.9)	0.46
≤ 7 drinks per week		
**Smoking Status**		** < 0.001**
Current smoker	2.0 (1.3, 3.1)	0.002
Non-smoker	reference	
**Physical Activity Outside of Current Job in Last Month**		** < 0.001**
No physical activity	1.7 (1.1, 2.6)	0.01
Some physical activity	reference	
**Fruit and Vegetable Consumption**		0.9
< 5 times per day	1.0 (0.6, 1.7)	0.9
≥ 5 or more times per day	reference	
**Frequent Mental Distress**		** < 0.001**
≥ 14 poor mental health days	2.2 (1.4, 3.4)	< 0.001
< 14 poor mental health days	reference	
**Body Mass Index (BMI)**		** < 0.001**
Obese (BMI ≥ 30)	3.3 (2.2, 5.1)	0.005
Overweight (25 < BMI ≤ 29)	1.9 (1.1, 3.1)	0.01
Normal (18.5 < BMI ≤ 25)	0.1 (0.01, 0.8)	0.005
Underweight (BMI ≤ 18.5)	reference	
**Problems with Medical Cost in Last 12 Months**		**0.004**
Could not afford needed care	1.7 (1.0, 2.8)	0.03
Afforded all needed care	reference	
**Personal Doctor**		** < 0.001**
No personal doctor	1.8 (1.1, 3.0)	0.03
Has personal doctor	reference	
**Most Recent Checkup**		** < 0.001**
Longer than 1 year ago	0.5 (0.3, 0.8)	0.005
Within last year (reference)	reference	
**Healthcare Insurance**		0.3
No healthcare insurance	1.3 (0.6, 2.9)	0.5
Has healthcare insurance	reference	
**Primary Care Health Professionals Shortage Area (HPSA)**		** < 0.001**
Resides in HPSA	1.6 (1.1, 2.3)	**0.01**
Does not reside in HPSA	reference	

### Descriptive analyses

Descriptive analyses were conducted using the surveyfreq procedure in SAS to account for the complex survey design of the BRFSS data [33]. The sampling weight (_LLCPWT), strata (_STSTR), and cluster (_PSU) variables were specified in each analysis [34]. The original sampling weights provided by the CDC were divided by three to account for aggregation of three years of surveys [34]. Weighted frequencies and hypertension prevalence were calculated with standard errors and 95% confidence levels, respectively. The full BRFSS dataset was not filtered to the target population, but instead domain analyses were conducted by calculating stratified tables based on respondents’ sex and age to ensure accurate calculation of standard errors and confidence intervals of weighted estimates [35].

### Predictor investigation

Model building followed a two-step process. First, all potential predictors from the conceptual model (Fig. 1) were assessed for simple associations with hypertension in univariable logistic regression models. Predictors were retained for further analysis if the *p*-value of the likelihood ratio test comparing the univariable model to a null model was < 0.20 [36]. All models accounted for complex survey designs by specifying the sampling weight, strata, and cluster variables using the surveylogistic procedure in SAS software version 9.4 [37].

The variables that had *p* < 0.20 in the 1st step were then assessed in a multivariable model using a manual backward selection process to identify a final parsimonious model. Covariates were removed from the model one-by-one based on *p*-values until all remaining variables were significant at a critical *p*-value of 0.05. At each step, the coefficients of remaining variables were compared before and after the removal of a covariate. If the coefficients changed by 20% or more, the variable being removed was considered a confounder and was retained in the model regardless of its statistical significance [36]. The predictive ability of the final model was assessed using a receiver operating characteristic (ROC) curve and area under the curve (AUC) [38].

## Results

### Descriptive analysis results

The weighted total number of women ages 18–44 years was 136,629 persons. Approximately 67% of the study population were aged 18–34 years, while the remaining 33% were aged 35–44 years. Most of the population was non-Hispanic white (78%), 5% was non-Hispanic American Indian, 4% was non-Hispanic black, and 13% were other races or ethnicities. Thirteen percent of the population were heavy drinkers, 16% were current smokers, 24% had no physical activity outside of work during the previous 30 days, 76% reported eating fruits or vegetables fewer than five times per day. Approximately 20% of the population reported frequent mental distress (FMD). After imputation of missing BMI data, most of the population was classified as either obese (33%) or overweight (25%). Approximately 43% of the population was unable to afford some needed medical care during the last 12 months, 20% had no personal healthcare provider, 32% did not have a healthcare checkup in the last 12 months, and 33% lived in a county designated as a primary care HPSA.

The overall prevalence of self-reported hypertension was 8.9% (95% CI = 7.4–10.4), but the prevalence varied widely across categories of some potential predictors. Notably, hypertension prevalence was disproportionately high among women who were American Indian or Other Race/Ethnicity (i.e., not white or black), current smokers, had no regular physical activity, had FMD, and were obese (Table 1). Of additional interest was that hypertension prevalence was lower among women who afforded all needed medical care in the last 12 months compared to women who could not afford some needed healthcare. Hypertension prevalence was also lower among those without a personal doctor compared to those with a personal doctor, and among women whose most recent healthcare checkup was more than 1 year ago compared to those who had a checkup within the last year (Table 1).

### Predictors of Hypertension

The following variables had significant univariable associations with hypertension, based on a critical *p*-value < 0.20, and were retained for assessment as potential predictors in the multivariable model: age, race, smoking status, physical activity, FMD, BMI, problems with medical costs, having a personal doctor, time of last recent checkup, and residence in a primary care HPSA (Table 2).

Based on the final multivariable logistic regression model, significant (*p* < 0.05) predictors of hypertension included age, FMD, BMI, time since last checkup, and residence in a primary care HSPA (Table 3). The odds of hypertension were higher among women who were aged 35–44 years, had FMD, were obese, and resided in a county designated as a primary care HPSA compared to women younger than 35 years, who did not have FMD, had a normal BMI, and did not reside in an HPSA, respectively. No confounders were identified. The AUC of the final model was 0.68.

**Table 3:** Results of the final multivariable binary logistic regression model investigating predictors of hypertension among women aged 18–44 years in North Dakota, 2017–2021

Level	Odds Ratio (95% Confidence Interval)	*p*-value
**Age**		
35–44	2.3 (1.6, 3.4)	** < 0.001**
18–34	reference	
**Frequent Mental Distress**		
≥ 14 poor mental health days	2.0 (1.3, 3.3)	**0.003**
< 14 poor mental health days	reference	
**Body Mass Index (BMI)**		** < 0.001**
Obese (BMI ≥ 30)	2.6 (1.7, 4.1)	** < 0.001**
Overweight (25 < BMI ≤ 29)	1.6 (1.0, 2.7)	**0.08**
Normal (18.5 < BMI ≤ 25)	0.1 (0.01, 0.8)	**0.03**
Underweight (BMI ≤ 18.5)	reference	
**Most Recent Checkup**		
Longer than 1 year ago	0.6 (0.4, 0.9)	**0.02**
Within last year (reference)	reference	
**Primary Care Health Professional Shortage Area (HPSA)**		
Resides in HPSA	1.8 (1.2, 2.6)	**0.003**
Does not reside in HPSA	reference	

## Discussion

This study investigated predictors of self-reported hypertension risk among women of reproductive age in North Dakota using data from the 2017, 2019, and 2021 BRFSS and county-level HPSA designations. The results suggest that among women of reproductive age in North Dakota, those who lived in primary care HPSAs or had FMD had significantly higher odds of hypertension compared to their counterparts who did not live in HPSA or have FMD, respectively. Similarly, those who were obese or 35–44 years old had significantly higher odds of hypertension compared to those who were not obese or were 18–34 years old, respectively. The findings of this study are useful for understanding risk factors of chronic hypertension among women of reproductive age in North Dakota and can be used to guide the development and implementation of programs aimed at reducing hypertension in this population.

The positive association between mental distress and hypertension is an important finding of this study. Self-reported mental distress is more prevalent among women and young adults than among men and older adults throughout the US, and the overall prevalence and disparities of FMD are increasing [39, 40]. Although mental health conditions, such as diagnosed depression, may have a direct biological association with the development of hypertension [41], the utility of self-reported mental distress in BRFSS data as a predictor of hypertension has not been adequately demonstrated. One study identified a univariable association between FMD and hypertension along with several other indicators of preconception health, but did not assess that association while controlling for other factors like BMI and age [42]. However, there is evidence that FMD is associated with a variety of other health conditions, including diagnosed mental disorders, indicating that self-reported mental distress is associated with diagnosable mental and physical health problems [43]. The results of this study indicate that among women of reproductive age in North Dakota, FMD is significantly associated with hypertension and may represent a modifiable risk factor.

Although cardiovascular risk factor prevalence tends to be higher among people residing in HPSAs, there is some evidence that the association between HPSAs and cardiovascular disease risk factors is eliminated when controlling for underlying sociodemographic characteristics [17]. Another study has reported that among uninsured populations, people residing in primary care HPSAs are less likely to achieve control of hypertension compared to those living in non-HPSAs [44]. The present study shows that among North Dakota women aged 18–44 years, the effect of HPSA is robust to the control of risk factors related to demographics, behaviors, and healthcare access and utilization. Women who did not have a healthcare checkup in the last year were also less likely to report diagnosed hypertension, which might suggest lower detection and reporting rates of existing hypertension compared to women who had checkups in the last year.

The associations between obesity, age, and hypertension are well documented [45–47]. Blood pressure naturally increases with age due to changes in arterial structure [45], and although women tend to have lower hypertension rates than men, blood pressure increases more rapidly in women after the age of 30 [46]. It is important for women at later ages to reduce the risk of developing hypertension by engaging in behaviors that lessen the risk of developing hypertension or avoiding behavioral risk factors. Obesity is also an independent risk factor of hypertension, but obesity rates increase with age, compounding the increased age-associated cardiovascular health risks [47]. Notably, women who were underweight in the present study had significantly lower odds of developing hypertension. This finding is consistent with those from other studies which reported that underweight was associated with lower risk of hypertension [48, 49]. However, there is evidence that underweight individuals have greater risk of cardiovascular disease than normal weight individuals, and one study reported that mortality rates among underweight people with severe hypertensions were significantly higher than among normal weight individuals [49, 50].

The unadjusted odds of hypertension were significantly higher among American Indian women than white women, but that effect was mediated by covariates in the multivariable model. This result is meaningful because of the health disparities that American Indian women in North Dakota face relative to the general population [20–24]. Based on this dataset, hypertension disparities among American Indian women of reproductive age are apparently mediated by some combination of demographic and behavioral risk factors, chronic conditions, and healthcare access or utilization. North Dakota public health officials should still consider racial disparities when designing programs to reduce hypertension in reproductive age women, but with the understanding that racial and ethnic disparities are mediated by other risk factors that were identified in this study.

### Study strengths and limitations

This is the first study to use BRFSS data to investigate predictors of hypertension among women of reproductive age in North Dakota, and the results provide an improved understanding of risk factors of hypertension in this population that are useful for guiding efforts to improve cardiovascular and maternal health in this population. The study provides novel evidence of a robust association between self-reported FMD and hypertension and strengthens our understanding of associations between primary care HPSAs and chronic hypertension in North Dakota. These findings are useful for the North Dakota Department of Health and Human Services to guide the implementation of programs to raise awareness and improve control of hypertensive disorders to improve health and reduce the risk of adverse pregnancy outcomes among women of reproductive age.

The use of BRFSS data means that hypertension classification was based on self-reporting and not direct measurement and hence the estimate of hypertension prevalence may be biased. Additionally, although FMD was a risk factor of hypertension in this study, that estimate of mental health is not specific to any condition. Finally, although HPSA was treated as a dichotomous variable in this study, the severity of primary care HPSAs are quantified by a numerical score that ranges from 0 to 25 [16]. In this study HPSA was used as a dichotomous variable to be consistent with other studies [17, 44, 51, 52]. Taken together, these limitations probably contribute to the relatively low predictive power of the final model. Suffice it to say that this study provides initial findings to guide future studies and provides useful information to guide health program planning.

## Conclusions

The odds of hypertension among women of reproductive age tend to be high among those with FMD, who reside in an HPSA, who are 35–44 years old, and who are obese. Women who did not have a checkup within the last year had lower odds of hypertension, probably indicating low detection and reporting rates in this segment of the population. These findings suggest that self-reported mental distress is a useful predictor of hypertension in this population, and further investigations should be conducted to determine whether reducing mental distress can reduce hypertension risk. The fact that high odds of hypertension were observed in HPSAs is important in guiding policy and public health service provision. Allocation of resources for public health programs intended to reduce chronic hypertension among women of reproductive age may need to prioritize HPSAs to reduce/eliminate these disparities and improve hypertension outcomes for all.

## Data Availability

The datasets generated and/or analyzed during the current study are available upon request to the North Dakota Department of Health and Human Services by emailing program staff at brfssnd@nd.gov.

## Abbreviations

ROC: Receiver Operating Characteristic
AUC: Area Under the Curve
OR: Odds Ratio
US: United States
HPSA: Health Professionals Shortage Area
BRFSS: Behavioral Risk Factor Surveillance System
NDDHHS: North Dakota Department of Health and Human Services
CDC: Centers for Disease Control and Prevention
BMI: Body Mass Index
FMD: Frequent Mental Distress
AI: American Indian/Alaskan Native
